# Characterization of the Biomass Degrading Enzyme GuxA from *Acidothermus cellulolyticus*

**DOI:** 10.3390/ijms23116070

**Published:** 2022-05-28

**Authors:** Neal N. Hengge, Sam J. B. Mallinson, Patthra Pason, Vladimir V. Lunin, Markus Alahuhta, Daehwan Chung, Michael E. Himmel, Janet Westpheling, Yannick J. Bomble

**Affiliations:** 1 Biosciences Center, National Renewable Energy Laboratory, 15013 Denver West Parkway, Golden, CO 80401, USA; neal.hengge@nrel.gov (N.N.H.); sam.mallinson@nrel.gov (S.J.B.M.); vladimir.lunin@nrel.gov (V.V.L.); petri.alahuhta@nrel.gov (M.A.); chung301@gmail.com (D.C.); mike.himmel@nrel.gov (M.E.H.); 2 Pilot Plant Development and Training Institute, King Mongkut’s University of Technology Thonburi, Bangkok 10150, Thailand; patthra.pas@mail.kmutt.ac.th; 3 Department of Genetics, University of Georgia, Athens, GA 30602, USA; janwest@uga.edu

**Keywords:** CAZymes, lignocellulosic biomass, enzyme synergy, cellulose, xylan, multifunctional enzymes

## Abstract

Microbial conversion of biomass relies on a complex combination of enzyme systems promoting synergy to overcome biomass recalcitrance. Some thermophilic bacteria have been shown to exhibit particularly high levels of cellulolytic activity, making them of particular interest for biomass conversion. These bacteria use varying combinations of CAZymes that vary in complexity from a single catalytic domain to large multi-modular and multi-functional architectures to deconstruct biomass. Since the discovery of CelA from Caldicellulosiruptor bescii which was identified as one of the most active cellulase so far identified, the search for efficient multi-modular and multi-functional CAZymes has intensified. One of these candidates, GuxA (previously Acel_0615), was recently shown to exhibit synergy with other CAZymes in C. bescii, leading to a dramatic increase in growth on biomass when expressed in this host. GuxA is a multi-modular and multi-functional enzyme from *Acidothermus cellulolyticus* whose catalytic domains include a xylanase/endoglucanase GH12 and an exoglucanase GH6, representing a unique combination of these two glycoside hydrolase families in a single CAZyme. These attributes make GuxA of particular interest as a potential candidate for thermophilic industrial enzyme preparations. Here, we present a more complete characterization of GuxA to understand the mechanism of its activity and substrate specificity. In addition, we demonstrate that GuxA exhibits high levels of synergism with E1, a companion endoglucanase from A. cellulolyticus. We also present a crystal structure of one of the GuxA domains and dissect the structural features that might contribute to its thermotolerance.

## 1. Introduction

In the last thirty years, significant progress has been made to optimize pretreatments and industrial cellulase preparations to improve biomass deconstruction. However, biomass deconstruction remains one of the main obstacles for efficient production of biofuels and biochemicals from lignocellulosic biomass [1,2]. Biomass recalcitrance has proven to be difficult to overcome no matter the conversion process; therefore, better, more robust, and more diverse biomass degrading enzymes are needed [3,4]. In general, the best-studied biomass degrading enzymes, those often used in industrial cellulase preparations, have a simple architecture that includes a catalytic domain and one or more carbohydrate binding domains separated by linker peptides [5]. These types of biomass degrading enzymes are the most commonly produced by cellulolytic microbes [6]. However, some cellulolytic bacteria rely instead on multi-modular and multi-functional biomass degrading enzymes with much more complex architectures [7,8]. In recent years, increasing numbers of these types of biomass degrading enzymes have been characterized due, in part, to the greater emphasis on thermophilic bacteria suitable for consolidated bioprocessing (CBP) [9,10].

One of these multi-modular and multi-functional biomass-degrading enzymes, CelA, shows remarkable activity on biomass and crystalline cellulose [7]. Some of this activity is thought to come from the complementary activities of its two glycoside hydrolases which promotes intramolecular synergy. Recently, chimeric enzyme constructs were successful in emulating some of this intramolecular synergy between GH domains in the same gene product [11]. Additionally, some of these enzymes show synergy with the more classical CAZymes with simpler architectures [7] in vitro and in vivo. For example, heterologous expression of the multifunctional GuxA (GH6/GH12) from A. cellulolyticus, in Caldicellulosiruptor bescii resulted in enhanced growth and cellulolytic activity of its exoproteome, but only in the presence of the endoglucanase, E1, from A. cellulolyticus [12,13]. E1 is a highly active and well characterized GH5 endoglucanase that was shown to exhibit high levels of synergy with several exoglucanases [12,13]. GuxA on the other hand contains an N-terminal GH6 domain linked to a CBM3 followed by a GH12 catalytic domain linked to a CBM2 on the C-terminal end. Of these domains, only the CBM3 exists in the native C. bescii exoproteome [13]. It was hypothesized that the addition of a potentially canonical exoglucanase (GH6) would improve the C. bescii secretome, which lacks a true and highly productive exoglucanase. In addition, the GH12 from GuxA might provide important endoglucanase xylanase activity given its proximity to the CBM2, which is known to also bind xylan [14]. In their study, Kim et al. found that despite improved growth of C. bescii when expressing GuxA, the activity of the exoproteome was not enhanced without the co-expression of E1. The conclusion from this study suggested that E1 is needed for GuxA to be fully active and to produce the endo/exo synergy found in the A. cellulolyticus secretome [12].

Given its unusual glycoside hydrolase combination, GuxA is a promising, but only partially characterized, biomass degrading enzyme. Here, we used C. bescii as an expression system for the multifunctional GuxA and characterized its substrate specificity, pH and temperature optima, as well as its activity on the model substrate, Avicel, and pretreated biomass. Additionally, we solved and analyzed the structure of the GH12 domain and suggest possible explanations for its thermostability.

## 2. Results and Discussion

### 2.1. Characterization of the pH and Temperature Range for GuxA Activity

**Figure 1:**
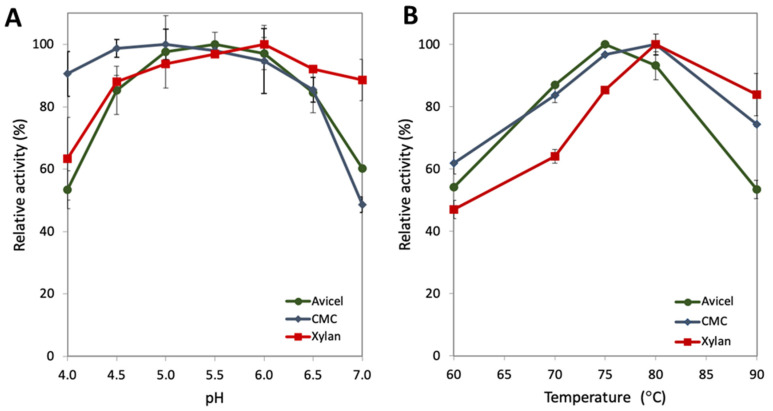
(**A**) Analysis of relative activity of GuxA on Avicel (green), CMC (blue) and xylan (red) at various pH conditions. (**B**) Analysis of relative activity GuxA on Avicel (green), CMC (blue) and xylan (red) at various temperatures.

Based on the glycoside hydrolase families found in GuxA, GH6 and GH12, several substrates were selected to determine its temperature and pH optima. Avicel is a model substrate for crystalline cellulose utilization. CMC, carboxymethyl cellulose, is an amorphous cellulose model substrate. Oat spelt xylan, is mixture of primarily xylose with some arabinose, glucose, and galactose to assess activity on xylan. Purified GuxA (Supplementary Figure S1) was tested on these substrates at temperatures ranging from 60 °C to 90 °C and pH values ranging from pH 4.0 to pH 7.0 (Figure 1). GuxA shows an optimal temperature range between 75 and 80 °C for all three substrates (Figure 1B), which is well above the optimal growth temperature of 55 °C for A. cellulolyticus. This temperature optimum is similar to that of other biomass degrading enzymes produced by this microorganism, including E1 [15,16]—a characteristic generally observed for other thermophilic enzymes [17]. The digestion of CMC occurred optimally at pH 5.0, but the difference in activity between pH 4.5 and pH 5.5 is not significantly different. This pH profile is similar across two units of pH, remaining above 80% of maximum activity (Figure 1A). The same result is seen when testing Avicel activity between pH 5.0 and pH 6.0 (maximum activity pH 5.5). Xylan digestion occurred optimally at pH 6.0. GuxA also retained over 80% of its activity on CMC between pH 4.0 and pH 6.5. Similar results were seen for GuxA activity on Avicel and xylan, however a sharp decrease in activity was observed at pH 4.0 (~53% for Avicel and ~63% for xylan). Additionally, of note is that ~89% of the xylanase activity was retained at pH 7.0 which is also true for other xylanases [18,19].

These measurements of activity in vitro suggest that this enzyme might represent a good partner in enzyme cocktails or in vivo applications for other hyperthermophilic enzymes, such as CelA and E1. The high tolerance for pH transitions, which is also required for use in combination with other enzymes is an encouraging feature. A wide range of pH tolerance is also desirable when the ultimate application requires use with cellulolytic thermophilic microorganisms that often acidify the culture medium during late stages of growth.

### 2.2. Activity of GuxA and Synergy with the Endoglucanase E1 on the Model Substrate Avicel

**Figure 2:**
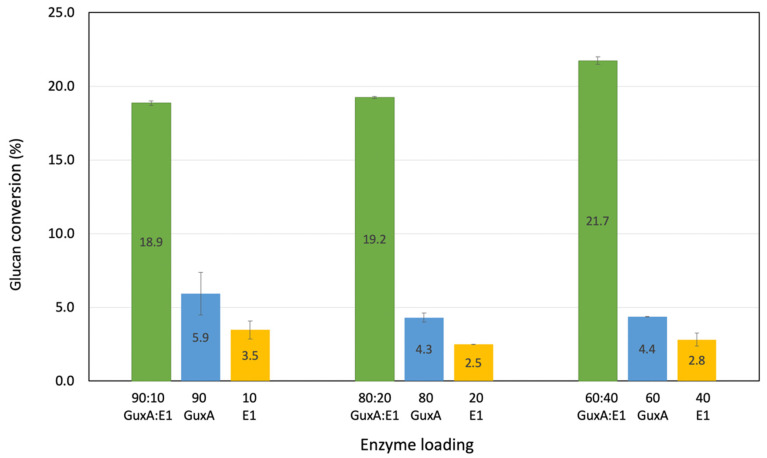
Investigation of the synergistic effects of GuxA with E1 on the deconstruction of Avicel using various mass ratios. Digestions were carried out for 72 h.

Kim et al. [13] previously reported that the co-expression of GuxA with E1 resulted in improved growth on cellulose. To better understand the potential synergistic effect suggested by this result, a series of relative protein loadings were tested to evaluate the synergy that may exist between the two enzymes. All reactions were carried out at the optimal temperature and pH, 75 °C and pH 5.5. It appears that GuxA alone shows low glucan conversion on Avicel after 72 h (glucan conversion no more than 6% for any individual loading even at the higher loadings of 13.5 mg/g substrate (Figure 2). This would indicate that the GH12 domain in GuxA is not able to make significant nicks in cellulose chains for the GH6 exoglucanase to act on. This observation is in agreement with the results reported in Kim et al. [13] where it was shown that the GH12 domain of GuxA exhibited primarily a xylanase activity with weak endoglucanase activity. However, significant levels of synergy were observed in cellulase mixtures of GuxA and E1 when acting on Avicel (Figure 2). The highest synergy was achieved after 72 h with a 60:40 molar ratio of GuxA and E1, respectively, corresponding to a 21.7% total glucan conversion. This indicates a high level of activity for the GH6 when combine with an efficient endoglucanase able to provide significant amount of cellulose chain ends.

### 2.3. Activity of GuxA and Synergy with the Endoglucanase E1 on Pretreated Biomass

**Figure 3:**
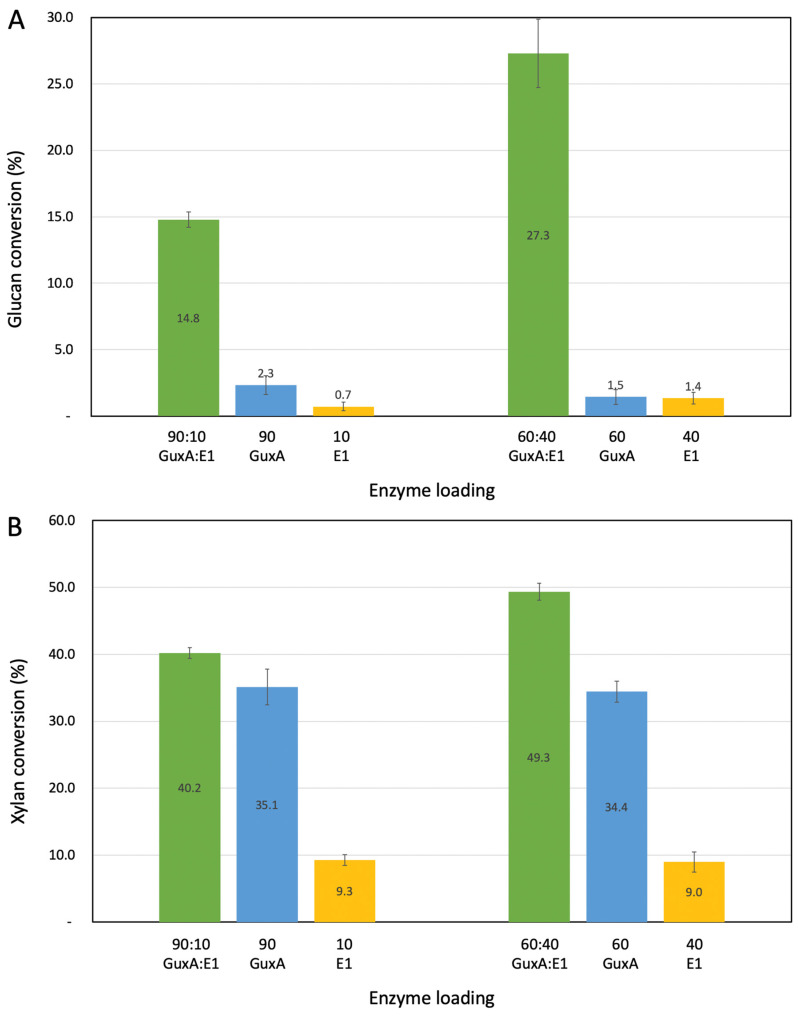
Investigation of the deconstruction of APCS: Glucan (**A**) and xylan (**B**) conversion using single enzymes and the two most extreme loadings of GuxA and E1 from Figure 2. Digestions were carried out for 72 h.

Alkaline-peroxide-pretreated biomass represents an appealing substrate for cellulosic biomass conversion as most of the biomass is stripped of its native lignin making it more amenable for deconstruction. Our APCS biomass includes primarily glucan (55.6%) and xylan (33.4%), with traces of lignin (3.9%); therefore, it is a relevant substrate for testing both cellulase and xylanase activity [20] on pretreated biomass. GuxA and E1 alone exhibit very low levels of glucan conversion with at most 2.3% of glucan release for GuxA with a high loading of 13.5 mg/g substrate. These conversion levels are even lower but similar to those observed on Avicel. However, high levels of conversions are observed with the addition of E1. In Figure 3, we considered the two best enzyme loadings previously determined on Avicel (Figure 2). Higher E1 loadings again produced a greater glucan conversion (Figure 3A) indicating the importance of the endoglucanase activity in this enzyme mix.

The xylan conversion results on the APCS substrate are very interesting. E1 alone as expected shows low levels of xylan conversion that is most likely the result of soluble sugars already present in the pretreated substrate. However, GuxA exhibited very high levels of conversion for a single enzyme with close to 35% of the xylan converted after 72 h. One interesting outcome is the fact that the conversion for GuxA alone reaches this maximum for the two different enzyme loadings considered (13.5 mg/g and 9.5 mg/g) which could be due to the complex nature of the substrate exposed at that point with branched xylans that the GH12 would not have activity towards. In enzyme mixes with GuxA and E1, surprisingly, the level of xylan solubilization on APCS was lower at higher GuxA loading (Figure 3B). However, this could be explained by the much higher glucan conversion at the 60:40 ratio which could help release/free up more accessible xylans for the enzymes to work on. Hence, more complete deconstruction of glucan is needed to increase overall xylan solubilization.

### 2.4. Structural Features of the GuxA GH12 Domain

**Figure 4:**
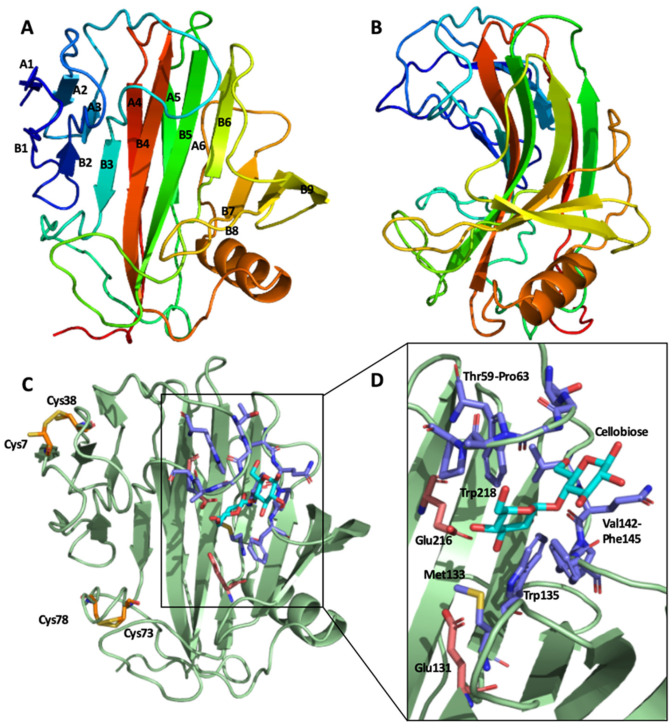
The crystal structure of the GuxA GH12 domain. (**A**) Viewed perpendicular to the β-jelly roll with β-strands labelled (N.B. strands A6 and B1, and parts of B7-B9 were not rendered as β-strands by PyMol). (**B**) The GH12 domain rotated 90° from A, viewed down the active site cavity. (**C**) Features of interest on the cellobiose bound structure—The two disulfides are shown in orange with cellobiose (cyan), the residues that coordinate it (blue) and the catalytic glutamate residues (light red) shown enlarged and reoriented in panel (**D**).

The structure of the GuxA GH12 domain was solved with and without cellobiose bound in the active site, to resolutions of 1.5 and 1.85 Å, respectively. The domain exhibits the classic GH12 β-jelly roll, with two curved β-sheets packed together-their concave face forming the active site-and one α-helix (Figure 4A,B). The conserved N-terminal disulfide bridge is present (between Cys7 and Cys38-all residue numberings follow the sequence of the crystal structure, Figure 4C), though the electron density indicates that this is partially reduced which might be an artefact of the X-ray diffraction experiment. A second disulfide bridge is also present, in two conformations, between residues Cys73 and Cys78 (Figure 4C). The active site cavity is lined with aromatic residues (Figure 4D) that accommodate the substrate without any significant change in structure at the local or whole-protein level—The Cα RMSD between the structures with and without cellobiose bound is 0.166 Å (Supplementary Figure S2). The reducing end of the bound cellobiose is sandwiched between two loops formed by residues Thr59-Pro63 and Val142-Phe145, and primarily contacts the peptide backbone atoms of these residues (Figure 4D). Glu131 and Glu216 are the catalytic residues (the nucleophile and the general acid/base, respectively), whereas the strictly conserved active site aspartate residue is at position 114.

The industrial relevance of GH12 enzymes has prompted several analyses of their structure-thermostability relationship [29,43]. Table 1 lists enzymes for which activity temperature optima are available and includes some of the features derived in this study from these published crystal structures that may play a role in thermostability. General trends for what contributes to high thermostability are suggested from a comparison of the different enzymes. Most striking is the hyperthermotolerance that seems to be conferred by having a large number of salt bridges, as per the GH12 enzymes from Pyrococcus furiosus and Thermotoga maritima, both of which have optimal activity at 100 °C. The other GH12 reported to have an optimum temperature of 100 °C, from Rhodothermus marinus, does not have an especially large number of salt bridges—instead, it appears to compensate for this with a pair of disulfides. The role of hydrophobic packing on thermostability amongst GH12 enzymes is well documented [37,43], and particular interest has been paid to the identity of the residues equivalent to alanine 35 in the T. reesei GH12 as single point mutants at this site have been shown to have a greater influence on enzyme stability than any other [29]. Specifically, larger, more hydrophobic side chains result in denser packing of the hydrophobic interior of the enzyme, stabilizing the folded state relative to unfolded [29]. In the case of the A. cellulolyticus GuxA GH12 domain, the leucine at this position probably makes a modest contribution to the enzyme’s thermal stability which, along with the extra disulfide bond, accounts for the thermotolerance of the enzyme. Comparison with the hyperthermotolerant GH12 enzymes suggests that the introduction of several additional salt bridges into the structure could raise the optimum temperature further, which could also be the case for its mesophilic and psychrophilic homologs.

## 3. Conclusions

Multi-modular and multi-functional biomass degrading enzymes represent a largely untapped resource for industrial cellulase preparations. GuxA from *Acidothermus cellulolyticus* is a robust CAZyme with a unique composition combining GH12 and GH6 domains that display high activity on pretreated biomass when combined with an endoglucanase. In addition to its expected thermotolerance, the observation that this enzyme retains greater than 80% of its activity across two pH units indicates that this protein may be well suited for industrial applications. The synergy experiments indicate that optimal biomass deconstruction by GuxA and E1 is obtained with a slight mass excess of GuxA, although significant synergy is still observed when GuxA is 90% of the enzyme loading, which is likely reflecting the reportedly greater endoglucanase activity of E1 compared with GuxA [13]. The activity of GuxA on xylan is also fairly high for a single gene product, which makes this enzyme of interest for inclusion in enzyme cocktails.

Analysis of the protein structure of the GH12 domain of GuxA provides some insight into its increased thermotolerance compared with other GH12 enzymes. Many lignocellulose-degrading microorganisms that produce both cellulose- and hemicellulose-degrading single domain enzymes also produce enzymes which tether together such activities. Understanding how these activities work together as individual enzymes and when tethered together remains a critical question in the field.

## 4. Materials and Methods

### 4.1. Construction of C. bescii Strains and Media Composition

Relevant plasmids used were electro-transformed into JWCB029 (ΔpyrFAΔldh::ISCbe4 Δcbe1ΔcelA) [44] cells as previously described [12]. Cultures, electro-pulsed with 0.5 µg of plasmid, were recovered in low osmolarity complex (LOC) growth medium at 75 °C. Recovery cultures were transferred to liquid low osmolarity defined (LOD) medium without uracil to allow selection of uracil prototrophs. Cultures were plated on solid LOD media to obtain isolated colonies, and total DNA was isolated from transformants using Quick-DNA kits (Zymo research, Irvine, CA, USA). PCR amplification using primers outside the gene cassette on the plasmid was used to confirm the presence of the plasmid with the gene of interest intact.

LOD was prepared using sterilized stock solutions including a 50× base salt solution containing 16.5 g of MgCl_2_, 16.5 g of KCl, 12.5 g of NH_4_Cl, 7 g of CaCl_2_ × 2H_2_O, and 0.68 g of KH_2_PO_4_ per liter, a 1000× trace minerals solution containing 1.5 g FeCl_2_ × 4H_2_O, 0.07 g ZnCl_2_, 0.1 MnCl_2_ × 4H_2_O, 0.006 g H_3_BO_3_, 0.19 CoCl_2_ × 6H_2_O, 0.002 g CuCl_2_ × 2H_2_O, 0.024 NiCl_2_ × 6H_2_O, and 0.036 Na_2_MoO_4n_ × 2H_2_O per liter, and a 2000× vitamin solution containing 0.04 g biotin, 0.04 g folic acid, 0.2 pyridoxine HCl, 0.1 thiamine HCl × 2H_2_O, 0.1 g riboflavin, 0.1 g nicotinic acid, 0.1 g D Ca D-pentothenate, 0.002 vitamin B12, 0.1 g p-aminobenzoic acid, and 0.1 lipoic acid. The stock solutions were added to reach a 1x final concentration to water and 1 g/L sodium bicarbonate, 1 g/L of L-cysteine HCl, and 5 g/L cellobiose were mixed into the medium before adjusting the pH to 6.8 using NaOH for titration. LOC was prepared identically but adding 1 g/L yeast extract and 2 g/L casein. Uracil can be used as nutritional selection marker and is added at 5 g/L when necessary.

### 4.2. Protein Expression in C. bescii and E. coli

C. bescii was used for extracellular expression of GuxA as follows: Frozen cultures transformed with the relevant expression plasmid were inoculated into serum vials containing 20 mL of low osmolarity defined growth medium (LOD) and incubated at 65 °C with mild agitation for approximately 24 h. Starter cultures were passaged at 2% (*v*/*v*) into serum vials each containing 100 mL of LOD and grown overnight under the same conditions. For each 10-L fermentation, 200 mL of total culture was used to inoculate 9.8 L of LOD medium containing 5 g/L cellobiose as the sole carbon source. Cultures were maintained at pH 6.8, 50 RPM agitation, 0.5 L/min N_2_ sparging, and temperature of 65 °C. A minimum of 16 h was needed to reach an OD_600_ of 0.6, at which cells were harvested through a polycap glass fiber filter (GE Healthcare Bio-Sciences, Marlborough, MA, USA) before being concentrated using hollow fiber concentrators containing a 10 kDa cutoff membrane (GE Healthcare, Piscataway, NJ, USA). The concentrator was also used to buffer exchange the concentrate into Buffer A (50 mM Tris, 250 mM NaCl, 10 mM imidazole, pH 8.0).

*E. coli* was used for intracellular expression of the GuxA GH12 domain. DNA sequences for the GuxA GH12 domain was codon optimized and cloned into a pET22b(+) vector (GenScript, Piscataway, NJ, USA). The sequence for a hexahistidine tag was placed at the C terminus of the constructs. The resulting construct was transformed in a BL21 (DE3) strain of Escherichia coli and cells were grown 4 h at 37 °C with 0.1 mM IPTG. Proteins were concentrated using Vivaspin spin columns with a molecular weight cutoff of 10,000 Da (GE Healthcare Life Sciences, Pittsburgh, PA, USA).

### 4.3. Protein Purification

Crude extracellular C. bescii concentrates (full length GuxA) or E. coli lysates (GH12 domain) were loaded onto and purified initially using a 5 mL HisTrap FF Crude column (GE Healthcare, Piscataway, NJ, USA) via FPLC (GE Healthcare AKTA Explorer, Piscataway, NJ, USA). The HisTrap column was initially equilibrated with Buffer A (50 mM Tris, 100 mM NaCl, and 10 mM imizadole, pH 8.0) followed by injection of the protein sample. After binding of the protein 100% of Buffer B (Buffer A with 200 mM imidazole) is used to elute the column and fractionate the sample. The samples were next prepared for size exclusion chromatography (SEC), which was completed using either a HiLoad 16/600 Superdex 200 (full length GuxA or HiLoad 16/600 Superdex 75 prep grade column (GH12 domain) (GE Healthcare, Piscataway, NJ, USA) depending on the molecular weight of the purified protein. The collected HIS elution fractions were combined and concentrated using a 10 kDa cutoff spin concentrator (Sartorius, Stonehouse, UK). Highly concentrated fractions were not further concentrated and instead divided into 5 mL samples for individual injections onto the size exclusion column. Samples were purified in this step using SEC Buffer (200 mM sodium acetate, 100 mM NaCl, 10 mM CaCl_2_, pH 5.5), which was used as the final storage buffer for these proteins. SEC fractions were collected and concentrated as necessary. Protein concentrations were determined using a Pierce BCA Protein Assay Kit (Pierce, Rockford, IL, USA).

### 4.4. Enzyme Activity Assays

Temperature and pH range experiments with GuxA were carried out using sugar release quantification on 10 g/L substrates including Avicel (PH101), carboxymethyl cellulose (CMC), and oat spelt xylan. All three substrates were obtained through Sigma Aldrich, St. Louis, MO. CMC and xylan digestions were carried out for 1 h at various temperatures and pH values to determine the optimum conditions for enzyme function, whereas Avicel digestions were carried out for 24 h. GuxA/E1 and β-D-glucosidase (Thermotoga maritima/Megazyme) were loaded at 15 mg (total enzyme)/g substrate and 1 mg/g substrate, respectively, in 1.5 mL centrifuge tubes. All assays were carried out in SEC buffer (pH 5.5 used for all temperature reactions and 75 °C was used for all pH reactions). The reaction tubes were kept at constant temperature using a water bath. Sugar release was measured using dinitrosalicylic acid (DNS). DNS solution was added to the reaction volume at a ratio of 2:1 and boiled for 5 min. Samples were then transferred to a 96-well plate and measured at OD_540_. Standard curves were generated using known quantities of glucose.

Multiple sugar release assays were utilized to determine various performance characteristics of individual enzymes and synergistic mixtures. The activities of GuxA and E1 individually and in mixtures were first tested using Avicel hydrolysis assays then APCS (Alkaline Pretreated Corn Stover). APCS was prepared using a mixture of 500 mg hydrogen peroxide per g of raw corn stover in deionized water with a final solids loading of 10%. 5 M NaOH was added until the pH of the slurry reached 11.5. The mixture was incubated at room temperature for 48 h in a shake incubator. The pretreated biomass was filtered using a coarse felt, and the solids were thoroughly washed with deionized water to neutral pH and stored at 4 °C. Avicel or APCS was loaded into 2 mL-screw-top vials to bring the final solids loading to 1% (w/v). An appropriate volume of SEC buffer was added along with the enzymes being tested and b-glucosidase from Thermotoga maritima to match the reaction volume to the correct solids loading (approximately 2 mL total volume). b-D-glucosidase was loaded at 1 mg/g cellulose, whereas all other enzymes of interest were loaded at 10 mg/g cellulose individually or total combined. All reactions were carried out in duplicate for 96 h at 75 °C in a rotisserie oven set at 12 RPM. 100 µL samples were collected at 72 h and diluted 10× before being 0.22 mM filtered into an HPLC vial for sugar analysis. Glucose, xylose, and cellobiose concentrations were determined using an HPX-87H 7.8 × 300 mm i.d. 9 mm column (BioRad, Hercules, CA, USA) using an isocratic flow of 0.01 N H_2_SO_4_ at 0.6 mL/min for a total run time of 27 m using standard protocols. A sample injection volume of 20 µL was used and a constant column/detector temperature of 55 °C was maintained. Coferm sugar standards from Absolute Standards (Hamden, CT, USA) were used to generate relevant calibration curves. The compositional analysis of the APCS substrate was 55.6% glucan, 33.4% xylan, 3.9% lignin, 1.2% galactan, 2.9% arabinan). More details about the compositional analysis and the pretreatment conditions can be found in Brunecky et al., 2017 [45].

### 4.5. Crystallization of the GuxA GH12 Domain

The crystal for the GH12 domain (MW 24 kDa) was obtained using sitting-drop vapor diffusion with Crystal Screen (Hampton Research, Aliso Viejo, CA, USA) using a 96-well plate. A 50 µL well solution was used with drops containing 1 µL well solution and 1 µL protein solution. The crystals were grown at 298 K in 10% PEG 8K as a precipitant in the presence of 0.2 M ZnCl_2_ and 0.1 M Na cacodylate buffer adjusted to pH 6.5; the protein was diluted to 5.86 mg/mL concentration in 20 mM acetic acid pH 5.0 buffer with 100 mM NaCl and 10 mM CaCl_2_. Before data collection, the crystal was briefly soaked in a drop of 50%/50% (v/v) paraffin/paratone mixture and flash-frozen in a cold nitrogen-gas stream at 100 K. The data collection was performed using a Bruker X8 MicroStar X-ray generator with Helios mirrors and a Bruker PLATINUM135 CCD detector. The wavelength was 1.542 Å. Data were indexed and processed with the Bruker Suite of programs v.2008.1-0 (Bruker AXS, Madison, WI, USA).

Molecular replacement was performed with MOLREP [46] using PDB ID 3B7M [47] as a starting model. Further model refinement and rebuilding was done using REFMAC5 [48] and Coot [49]. Final model was refined to R/Rfree factors of 0.131/0.171 with 98.32% Ramachandran favored, 1.68% allowed, and 0% outliers. The model was deposited to the PDB with accession code 7MKR. Crystals described above were used to obtain complex with cellobiose by soaking for 24 h with the excess of cellobiose powder added to the crystallization drop. Diffraction data were collected at the same X-ray source. A cellobiose molecule was found and modeled in the active site groove. The model was refined to the final R/Rfree factors of 0.134/0.165 with 97.48% Ramachandran favored, 1.68% allowed, and 0.84% outliers. Crystallographic Table 1 can be found in SI (Table S1). The model was deposited to the PDB with accession code 7MKS. Structure images were generated using PyMol [50] and RMSD calculations, and intramolecular bond analysis was performed using Yasara [51].

## Figures and Tables

**Table 1:** Comparison of structural features of GH12s and their temperature optima. Where more than one PDB structure was available, the highest resolution structure with no mutations was chosen. The Ala35 equivalent column refers to the residue that is present at the position corresponding to the T. reesei GH12 alanine 35.

GH12 Source Organism	PDB	Cα RMSD w/7MKR (Å)	Temperature Optimum (°C)	Hydrogen Bonds	Hydrophobic Interactions	Salt Bridges	Disulfide Bridges	Ala35 Equivalent
Acidothermus cellulolyticus	7MKR	0.000	80	176	1034	3	2	L
Pyrococcus furiosus	3VGI [21]	1.397	100 [22]	212	1652	20	0	Y
Bacillus licheniformis	2JEM [23]	1.37	60 [24]	187	1206	9	0	H
Rhodothermus marinus ITI378	2BWA [25]	0.967	100 [26]	173	1157	9	2	V
Streptomyces lividans	2NLR [27]	1.188	60 [28]	171	1054	5	2	A
Streptomyces sp 11AG8	1OA4 [29]	1.237	≥60 [29]	174	1022	7	2	V
Thermotoga maritima	3AMH [30]	1.425	100 [31]	202	1624	20	0	F
Uncultured bacterium	3WX5 [32]	0.98	90 [32]	173	1211	9	2	I
Aspergillus aculeatus F-50	5GM4 [33]	1.27	50 [33]	169	1044	4	1	V
Aspergillus aculeatus KSM 510	3VL8 [34]	1.344	50 [35]	159	1037	2	1	V
Aspergillus niger CBS 120.49/N400	1KS4 [36]	1.324	60 [37]	141	1002	4	1	V
Aspergillus niveus PR-2	4NPR [38]	1.279	60 [39]	156	1061	2	1	F
Hypocrea schweinitzii ATCC 66965	1OA3 [29]	1.253	50 [29]	168	1032	3	1	S
Trichoderma harzianum IOC-3844	4H7M [40]	1.293	48 [41]	166	1044	4	1	V
Trichoderma reesei QM9414	1H8V [42]	1.264	50 [29]	157	991	3	1	A

## Data Availability

The data presented in this study are included in this published article/supplementary file or available from the PDB at 10.2210/pdb7MKR/pdb and 10.2210/pdb7MKS/pdb. Further inquiries can be directed to the corresponding author.

## Supplementary Materials

The following supporting information can be downloaded at: https://www.mdpi.com/article/10.3390/ijms23116070/s1.
