# Supplementary material for: Characterization of the Biomass Degrading Enzyme GuxA from Acidothermus cellulolyticus
Source: Int J Mol Sci. 2022 May 28;23(11):6070. doi: 10.3390/ijms23116070 (PMC9181691; doi:10.3390/ijms23116070)
Supplement: Supplementary file 1 [file ijms-23-06070-s001.zip › ijms-1683058-supplementary.pdf]

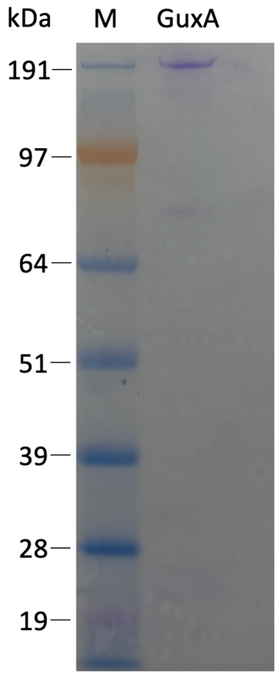

Figure S1: SDS-PAGE gel stained with Coomassie stain showing the purification product isolated after Ni-NTA affinity and HiLoad 16/600 Superdex 200 size exclusion purification in lane 2, Invitrogen™ SeeBlue™ Plus2 Pre-stained Standard in lane 1.

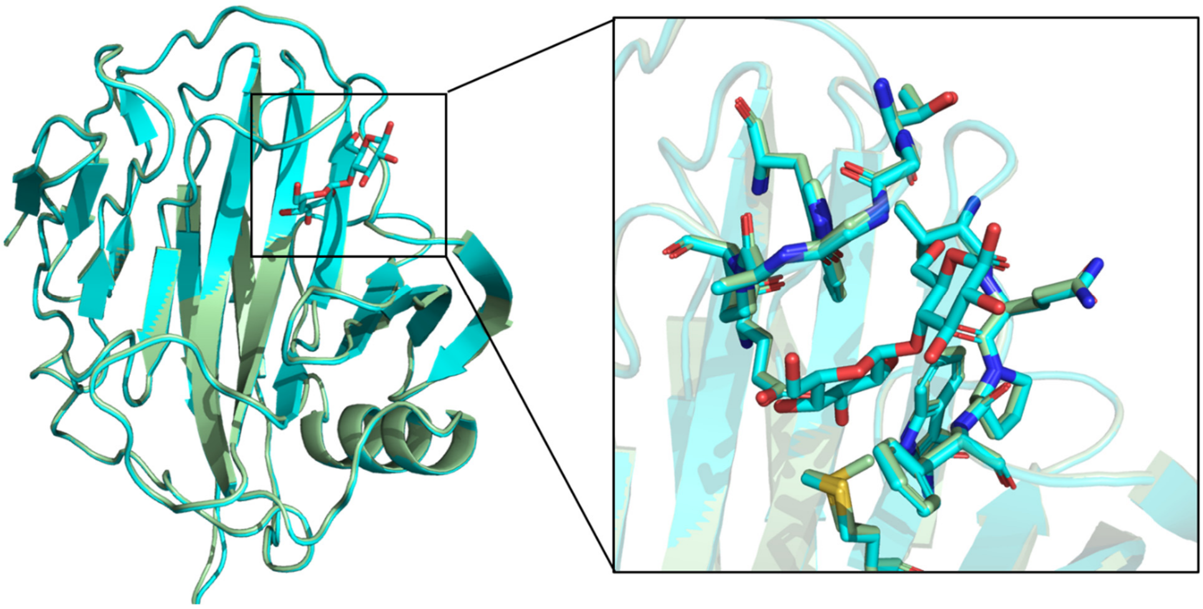

Figure S2. Alignment of GH12 with (green) and without cellobiose (cyan). Inset is a zoomed in view of the cellobiose with residues within 4 Å of the ligand represented as sticks. The representations together show the absence of any rearrangement of the structure on binding of cellulose at the whole-enzyme level and at the level of the residues that are directly involved in binding the substrate.

Table S1: Crystallography data collection and refinement statistics.

**Data collection and refinement statistics.**		
	**GuxA GH12 domain – 7MKR**	**GuxA GH12 domain bound to cellobiose – 7MKS**
**Data collection**		
Space group	P 31 2 1	P 31 2 1
Cell Dimensions		
a, *b*, *c* (Å)	66.2 66.2 127.9	66.2 66.2 127.2
α, β, γ (°)	90 90 120	90 90 120
Resolution range	27.96 - 1.5 (1.53 - 1.50)	34.09 - 1.85 (1.89 - 1.85)
R-merge	0.052 (0.329)	0.0380 (0.2920)
CC1/2	0.997 (0.654)	0.998 (0.755)
Mean I/sigma(I)	10.5 (2.3)	17.2 (2.8)
Completeness (%)	99.7 (97.4)	99.7 (95.5)
Multiplicity	1.9 (1.8)	1.9 (1.7)
**Refinement**		
Resolution	27.98 - 1.5 (1.539 - 1.5)	34.12 - 1.85 (1.898 - 1.850)
Reflections used in refinement	49887 (3567)	26747 (1856)
R-work/R-free	0.1291 (0.3270)/0.1711 (0.3740)	0.1328 (0.2160)/0.1649 (0.2460)
Number of atoms	2438	2271
macromolecules	1908	1897
ligands	49	45
solvent	481	329
Average B-factor	14.9	15.8
macromolecules	9.7	13.2
ligands	23.4	18.7
solvent	34.6	30.1
RMS (bonds, Å)	0.018	0.015
RMS (angles, °)	2.039	1.898
